# Relationships between sympathetic markers and heart rate thresholds for cardiovascular risk in chronic heart failure

**DOI:** 10.1007/s00392-022-02028-9

**Published:** 2022-05-12

**Authors:** Guido Grassi, Gino Seravalle, Jennifer Vanoli, Rita Facchetti, Domenico Spaziani, Giuseppe Mancia

**Affiliations:** 1 Clinica Medica, Department of Medicine and Surgery, University of Milano-Bicocca, Via Pergolesi 33, 20052 Milan, Monza Italy; 2 IRCSS Istituto Auxologico Italiano, Milan, Italy; 3 Unità Operativa Complessa Di Cardiologia, Magenta Hospital, Milan, Magenta Italy; 4 University Milano-Bicocca, Milan, Italy

**Keywords:** Heart failure, Heart rate, Sympathetic activity, Sympathetic nerve traffic, Plasma norepinephrine, Cardiovascular morbidity, Cardiovascular mortality

## Abstract

**Background:**

Results of recent clinical trials have shown that in heart failure (HF) heart rate (HR) values > 70 beats/minute are associated with an increased cardiovascular risk. No information is available on whether the sympathetic nervous system is differently activated in HF patients displaying resting HR values above or below this cutoff.

**Methods:**

In 103 HF patients aged 62.7 ± 0.9 (mean ± SEM) years and in 62 heathy controls of similar age we evaluated muscle sympathetic nerve traffic (MSNA, microneurography) and venous plasma norepinephrine (NE, HPLC assay), subdividing the subjects in different groups according to their resting clinic and 24-h HR values.

**Results:**

In HF progressively greater values of clinic or 24-h HR were associated with a progressive increase in both MSNA and NE. HR cutoff values adopted in large scale clinical trials for determining cardiovascular risk, i.e., 70 beats/minute, were associated with MSNA values significantly greater than the ones detected in patients with lower HR, this being the case also for NE. In HF both MSNA and NE were significantly related to clinic (*r* = 0.92, *P* < 0.0001 and *r* = 0.81, *P* < 0.0001, respectively) and 24-h (*r* = 0.91, *P* < 0.0001 and *r *= 0.79, *P* < 0.0001, respectively) HR. The behavior of sympathetic markers described in HF was specific for this clinical condition, being not observed in healthy controls.

**Conclusions:**

Both clinic and 24-h HR values greater than 70 beats/minute are associated with an increased sympathetic activation, which parallels for magnitude the HR elevations. These findings support the relevance of using in the therapeutic approach to HF drugs exerting sympathomoderating properties.

**Graphical abstract:**

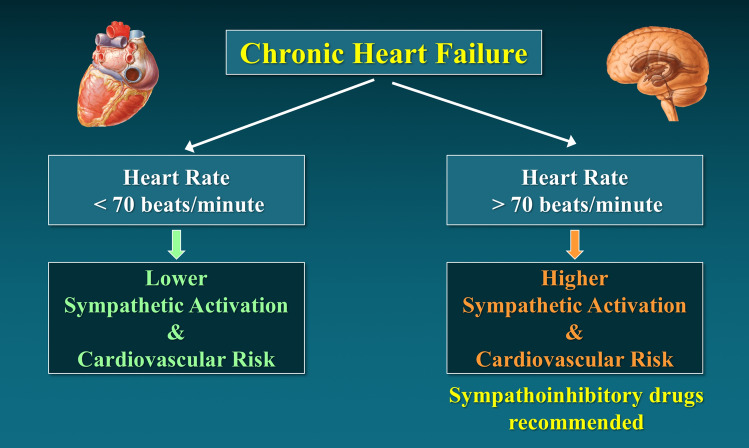

Several studies have unequivocally shown that in patients with chronic heart failure (HF) an elevated resting heart rate (HR) predicts the occurrence of fatal and non-fatal cardiovascular outcomes independently from other known risk factors [1–9]. Data collected in the context of the Systolic Heart Failure Treatment with the *I*_*t*_ inhibitor ivabradine Trial (SHIFT) have additionally shown that in HF the cutoff value of HR above which cardiovascular risk is increased is positioned at 70 beats/minute [4, 5]. While similar cutoff values of HR have been applied in other four large scale studies [7–10], a lower one (60 beats/minute) was applied in the morBidity–mortality EvAlUaTion of the *I*_f_ inhibitor ivabradine in patients with coronary disease and leFt ventricULar dysfunction (BEAUTIFUL) trial [6]. Among the different mechanisms potentially responsible for the above findings, an important one is represented by the stimulation of the sympathetic influences to the heart, given the evidence that an elevated HR reflects an activation of the adrenergic cardiovascular drive [11, 12]. This sympathoexcitation has been repeatedly shown in HF to be directly and independently related to cardiovascular morbidity and mortality [13–18]. However, no information is so far available on whether the sympathetic nervous system is differently activated in HF patients displaying resting HR values above or below the above mentioned cutoff. This information is crucial to determine whether more or less elevated HR values are capable to reflect in HF a different degree of sympathetic activation. The present study was designed to address this issue by assessing sympathetic drive in a large sample of HF patients displaying resting HR values above or below the above mentioned cutoff. This was done by assessing sympathetic cardiovascular drive via venous plasma norepinephrine (NE) assay and microneurographic recording of muscle sympathetic nerve traffic (MSNA). The same analysis was additionally performed in healthy subjects to determine whether the behaviour of HR as adrenergic marker seen in HF was specific to this clinical condition or rather it was also common to the general population.

## Methods

### Population

The study population consisted of 103 HF patients of both genders (78 males, 25 females) with an age range between 42 and 70 years and of 62 healthy individuals (48 males, 14 females) with superimposable age. For both groups the evaluation was done retrospectively and it was based on the detection of a HR value below or above 70 beats/minute at the office visit performed the day preceding the microneurographic nerve traffic recording session made in the frame of different investigations carried out between 2016 and 2021. All individuals included in the study were normotensive and in sinus rhythm and no individual had a history of myocardial infarction in the 12 months before the study or clinical or laboratory evidence of valvular heart disease, renal insufficiency, hypertension, diabetes mellitus or any other condition (except HF in the HF group) known to affect autonomic modulation of the cardiovascular system [18]. While healthy controls were pharmacologically untreated, HF patients were under stable medication treatment for at least 8 weeks before performing the evaluation. Treatment regimen included loop diuretics, angiotensin-converting enzyme inhibitors, angiotensin II receptor antagonists, angiotensin receptor neprilysin inhibitors, antialdosterone drugs, nitrates and calcium channel blockers in various combinations and dosages. Beta-blocking agents and ivabradine, if present, were withdrawn 5–7 days before the study proper. Both patients and healthy controls were evaluated on an outpatient basis and gave their written consent to the study after being informed of its nature and purpose. The study protocol was approved by the Ethics Committee of one of the institutions involved.

### Measurements

Measurements included body mass index, sphygmomanometric and beat-to-beat finger systolic and diastolic blood pressure via a validated instrument (Finapres, Ohmeda 2003, Englewood, FL, USA), HR (EKG), respiration rate (pneumotacograph), and oxygen saturation (pulse oxymeter, Nellcor, Medtronic, USA). They also included (1) MSNA via the microneurographic technique [11, 12, 16, 18], (2) venous plasma NE via high-performance liquid chromatography with electrochemical detection (Machery-Nagel ET 200/4 Nucleosil 100–5 C18 column, Machery-Nagel, and Waters 460 electrochemical detector; Waters GmbH, Eschborn, Germany) [19], (3) beta-type brain natriuretic peptide (BNP) via the Elecys 2010 analyzer from Roche Diagnostic (Mannheim, Germany) [20] in the group of HF patients and (4) an echocardiographic assessment of the end-diastolic and end-systolic left ventricular internal diameters, left atrial diameters and left ventricular ejection fraction, measured from the four-chamber apical projection using the product area times length [21]. Echocardiographic data also included mitral flow [early diastolic peak flow velocity (E wave) and late diastolic peak flow velocity (A wave)] and flow at the left ventricular outflow tract values. An EKG-Holter monitoring was performed during the 24 h in both HF patients and healthy controls.

Simultaneous MSNA, beat-to-beat HR and blood pressure recordings were digitized with a sampling frequency of 1000 Hz (PowerLab Recording System Model ML870 8/30; AD Instruments, Bella Vista, New South Wales, Australia). MSNA was quantified over a 30-min period as bursts incidence over time (bursts/min) [11, 12, 22]. This quantification was shown to be highly reproducible, that is to differ by only 4.3% when assessed on two separate occasions [22].

### Protocol and data analysis

All participants were examined in the morning after a light breakfast and an overnight abstinence from alcohol and coffee consumption. They were asked to assume the supine position, after which 3 sphygmomanometric blood pressure and HR (palpatory method, radial artery) values were obtained. Following the BP and HR measurements, the patients were fitted with an intravenous cannula and the devices to measure finger BP and to record an EKG. Blood samples were taken 30 min after positioning the venous cannula. A microelectrode was then inserted into a peroneal nerve to obtain MSNA, which was recorded together with finger BP and the EKG during a 30-min period. Data were collected in a semidark and quiet room kept at a constant temperature of 20 °C–22 °C. As mentioned above, the study had a retrospective nature and included data collected and already blindly analyzed for other microneurographic studies not related to the objective of the present investigation. We included in the present analysis all patients examined in previous studies who fulfilled the criteria of enrollment mentioned above. This allowed to avoid any potential bias in data analysis. Values from individual participants were averaged for each patients group (see below) and expressed as means ± SEM. The two study populations were subdivided into different groups (four for HF patients and three for controls) according to the clinic HR values displayed at rest by the subjects. Comparisons between groups were made by 2-way ANOVA, using the Student *t* test for unpaired observations or by chi-square statistic to determine their differences. The Pearson correlation coefficient was used to determine the relationships between resting MSNA and other parameters, a *P* < 0.05 being taken as the minimal level of statistical significance. All statistical analyses were performed by SAS software version 9.4 (SAS Institute Inc, Cary, NC, USA).

## Results

### HF patients

**Fig. 1:**
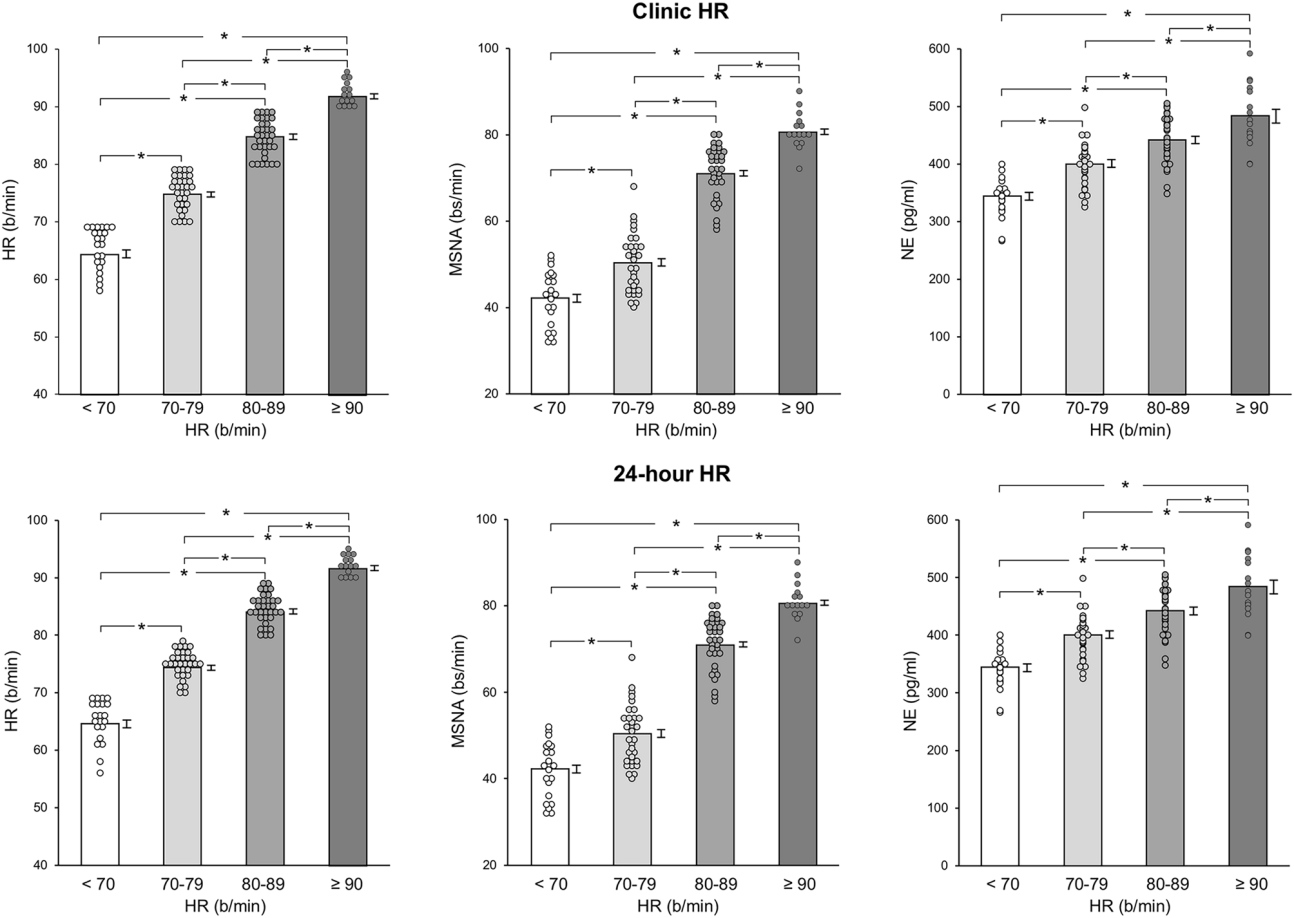
Upper panels: individual and average values (± SEM) of clinic heart rate (HR), muscle sympathetic nerve traffic (MSNA), and venous plasma norepinephrine (NE) in the groups of patients with heart failure with resting clinic heart rate < 70 beats minute, between 70 and 79 beats/minute, between 80 and 89 and > 90 beats/minute. Lower panels: individual and average values (± SEM) of 24-h (24 h) heart rate (HR), muscle sympathetic nerve traffic (MSNA), and venous plasma norepinephrine (NE) in the groups of patients with heart failure with resting 24-h heart rate < 70 beats minute, between 70 and 79 beats/minute, between 80 and 89 and > 90 beats/minute. Bs/min indicates bursts/minute. Asterisks (*P < 0.05, **P < 0.01) refer to the statistical significance between groups

As shown in Table 1 the 4 groups of HF patients characterized by resting clinic HR values below 70, between 70 and 79, 80 and 89 and above 90 beats/minute displayed a similar gender distribution and a superimposable age. This was the case for body mass index, systolic and diastolic BP, serum hemoglobin, serum glucose, plasma creatinine and estimated glomerular filtration rate. Left ventricular ejection fraction was similarly reduced in the 4 groups, which showed also a similar New York Heart Association functional class distribution, a similar number of HF cases of ischemic or idiopathic nature and a similar distribution of patients with preserved, midrange and reduced ejection fraction. Similar increases in BNP values were detected in the 4 groups. Individual and average clinic HR values assessed in the 4 HF groups are shown in Fig. 1, which also displays individual and average data related to 24-h HR Holter monitoring, MSNA and venous plasma NE. As expected, in HF patients resting HR values were progressively and significantly increased from the group with an HR below 70 beats/minute to the ones displaying HR between 70 and 79, 80 and 89 and above 90 beats/minute (Fig. 1, left upper panel). This was the case also for 24-h HR values (Fig. 1, left lower panel). More importantly, although consistent interindividual differences were detected, patients with clinic HR values greater than 70 beats/minute were characterized by MSNA and NE values significantly increased as compared to those found in HF with HR lower than 70 beats/minute (Fig. 1, upper middle and right panels). The increase was progressively greater for magnitude from the group with HR between 70 and 79 beats/minute to the ones with HR between 80 and 89 and above 90 beats/minute, respectively. A similar behavior was found when HR values were assessed during the 24-h Holter monitoring (Fig. 1, lower middle and right panels).

**Table 1:** Demographic, hemodynamic and clinic characteristics of heart failure patients with different clinic heart rate (HR) values

Variable	HR < 70 b/min (*n* = 22)	HR 70–79 b/min (*n* = 32)	HR 80–89 b/min (*n* = 34)	HR > 90 b/min (*n* = 15)
Age (years)	63.4 ± 2.9	62.8 ± 2.2	62.4 ± 2.2	62.5 ± 3.2
Gender (M/F, *n*°)	15/7	26/6	27/7	10/5
BMI (Kg/m^2^)	24.4 ± 0.5	24.2 ± 0.3	24.6 ± 0.3	24.9 ± 0.6
Systolic BP (mmHg)	123.4 ± 3.5	122.8 ± 3.3	121.2 ± 3.2	119.5 ± 4.1
Diastolic BP (mmHg) Heart rate (b/min)	76.2 ± 2.4 65.4 ± 0.8	75.5 ± 2.2 75.1 ± 0.6**	76.0 ± 2.1 84.6 ± 0.7**,††	76.3 ± 3.0 92.2 ± 0.8**,††^,#^
LVEF (%)	35.2 ± 0.8	33.4 ± 0.7	32.9 ± 0.6	31.8 ± 1.0
NYHA I/II/III/IV (*n*°)	2/13/6/1	1/19/10/2	0/19/12/3	0/4/7/4
Dilat/Ischemic (n°)	6/16	9/23	10/24	5/10
HFpEF/mEF/rEF (*n*°) BNP (pg/ml)	3/10/9 216.7 ± 17	1/13/18 248.4 ± 13	1/14/19 245.1 ± 12	0/4/11 269.8 ± 19
Hemoglobin (g/dl)	13.3 ± 2.2	12.9 ± 2	13.0 ± 1.8	13.1 ± 2.5
Glucose (mg/dl)	88.5 ± 8	89.7 ± 5	89.2 ± 5	91.1 ± 9
Creatinine (mg/dl)	0.91 ± 0.2	1.2 ± 0.3	1.1 ± 0.2	1.3 ± 0.4
eGFR (ml/min/173 m^2^)	73.2 ± 8	73.7 ± 6	76.1 ± 7	77.4 ± 11
Resp rate (br/min)	16.9 ± 1.2	17.1 ± 0.9	17.0 ± 1.0	17.5 ± 1.4
N° drugs/day	3.5 ± 0.2	3.7 ± 0.1	4.0 ± 0.1	4.3 ± 0.3*, †
ACEI/ARB/ARNI (%)	96	100	100	100
Loop diuretics (%)	100	100	100	100
Nitrates (%)	50	52	58	61
Others (%) Ivabradine (%)	52 0	55 6	57 9	56 13
B blockers (%)	86	88	100	100

Data are shown as mean ± SEM or percent (%) values. *M* Males, *F *females, *BMI *body mass index, *BP *blood pressure, *LVEF* left ventricular ejection fraction, *NYHA* New York Heart Association, *Dilat* Dilated, *HfpEF *heart failure with preserved ejection fraction, *mEf *mid-range ejection fraction, *rEF *reduced ejection fraction, *BNP *brain natriuretic peptide, *eGFR *estimated glomerular filtration rate, *resp* respiration, *b/min* beats/ minute, *br *breaths. *P < 0.05 **P < 0.01vs HR < 70, ^†^*P* < 0.05 ^††^*P* < 0.01 vs HR 70–79, ^#^*p* > 0–01 vs 80–89. Note: Beta-blockers and Ivabradine withdrawn 5–7 days prior to the study. “Others” include digitalis compounds, calcium channels blockers and antialdosterone drugs

**Fig. 2:**
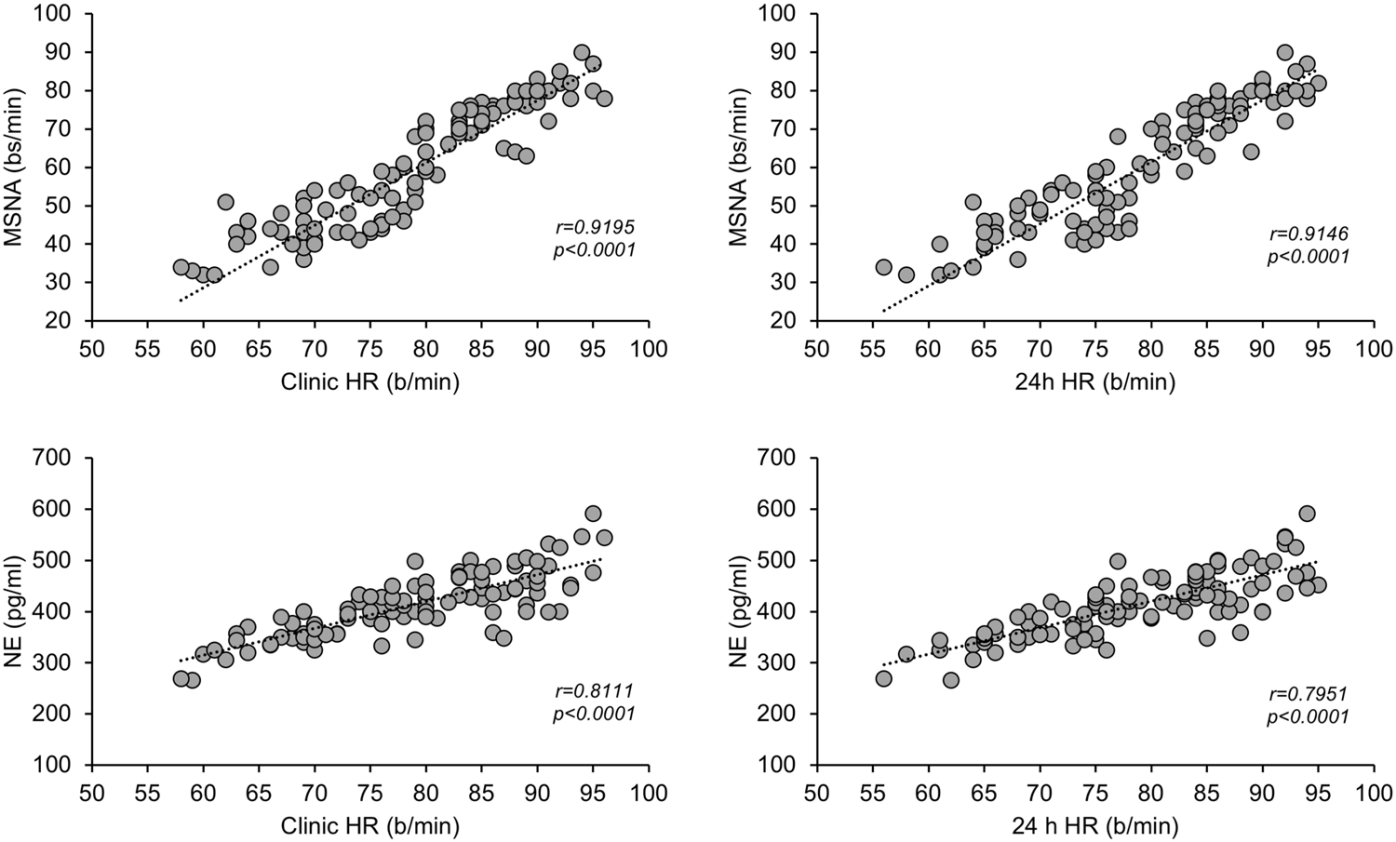
Left: regressing clinic heart rate (HR) on muscle sympathetic nerve traffic (MSNA), expressed as bursts frequency over time (bursts per minute, bs/min, upper) and plasma norepinephrine (NE, lower) in 103 heart failure patients. Correlation coefficients (*r*) and *P* values are shown. Right: regressing 24-h HR on MSNA, expressed as bursts frequency over time (bursts per minute, upper) and venous plasma NE (lower) in the same 103 HF patients displayed on the left panels. Correlation coefficients (*r*) and *P* values are shown

As shown in the left upper and lower panels of Fig. 2, in the group as a whole MSNA and plasma NE showed a highly significant relationship with clinic HR, the correlation being closer for MSNA than for NE. Similar relationships were found when 24-h HR values were considered (Fig. 2, right upper and lower panels). When correlations were sought separately in the 4 different groups of HF patients MSNA was always significantly related to HR, while NE only in the 2 groups of patients with greater HR values, i.e., between 80 and 89 and above 90 beats/minute (data not shown). In the group of HF patients as a whole both MSNA and NE showed significant direct relationships with left ventricular ejection fraction (*r *= 0.35 and *r* = 0.24, *P* < 0.001 and *P* < 0.05, respectively).

### Healthy controls

Table 2 reports the main demographic, anthropometric, hemodynamic, and biochemical characteristics of the 3 groups of healthy control subjects displaying different resting clinic HR values, i.e., below 70, between 70 and 79 and above 80 beats/minute, respectively. The 3 groups showed superimposable values of the various variables examined, including left ventricular ejection fraction, which was always within the normal range.

**Table 2:** Demographic, hemodynamic and clinical characteristics of healthy subjects with different clinic heart rate (HR) values

Variable	HR < 70 b/min (*n* = 20)	HR 70–79 b/min (*n* = 23)	HR > 80 b/min (*n* = 19)
Age (years)	64.4 ± 2.5	64.0 ± 2.4	63.6 ± 2.8
Gender (M/F) (*n*°)	15/5	17/6	16/3
BMI (Kg/m^2^)	24.0 ± 0.8	24.2 ± 0.6	24.5 ± 0.9
SBP (mmHg)	129.5 ± 3.1	130.1 ± 3.0	131.4 ± 3.3
DBP (mmHg) Heart Rate (b/min)	80.0 ± 2.6 63.9 ± 0.9	79.6 ± 2.5 75.1 ± 0.6**	80.6 ± 2.8 83.4 ± 0.7**,††
LVEF (%)	62.4 ± 2.2	63.4 ± 2.0	62.8 ± 2.5
Hemoglobin (g/dl)	14.0 ± 0.4	13.7 ± 0.3	13.8 ± 0.4
Glucose (mg/dl)	86.0 ± 9.0	87.5 ± 8.1	88.2 ± 9.8
Creatinine (mg/dl)	1.0 ± 0.2	0.95 ± 0.3	0.98 ± 0.3
eGFR (ml/min/173 m^2^)	89.4 ± 5	87.1 ± 6	88.0 ± 7
Resp rate (br/min)	16.5 ± 1.3	16.8 ± 1.0	16.3 ± 1.4
N° drugs/day	0	0	0

Data are shown as mean ± SEM or percent (%) values. **P < 0.01 vs HR < 70, ^††^*P* < 0.01 vs HR 70–79. Other symbols and abbreviations as in Table 1

**Fig. 3:**
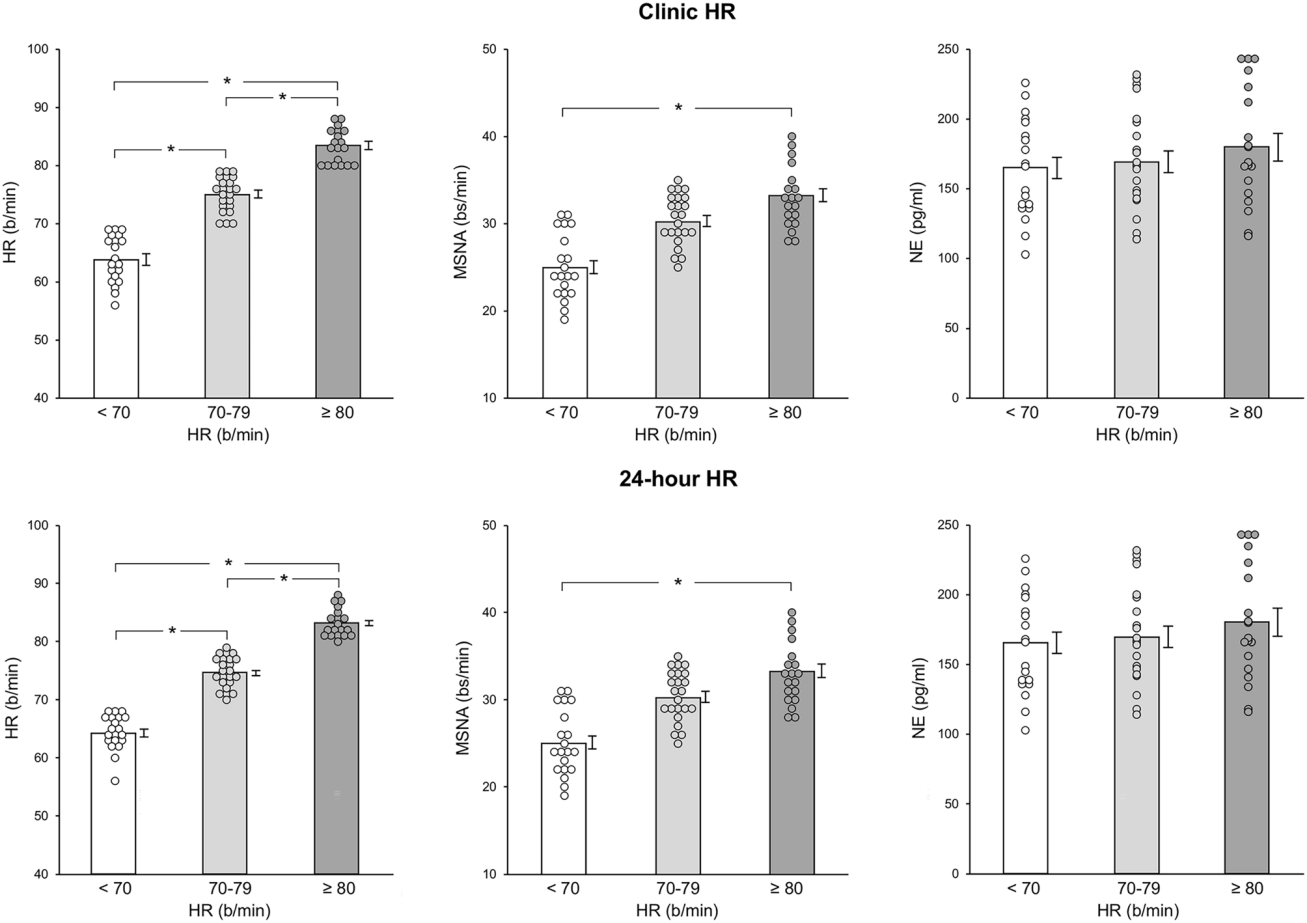
Upper panels: individual and average values (± SEM) of clinic heart rate (HR), muscle sympathetic nerve traffic (MSNA), and venous plasma norepinephrine (NE) in the groups of 62 healthy control subjects with resting clinic heart rate < 70 beats minute, between 70 and 79 beats/minute > 80 beats/minute. Lower panels: individual and average values (± SEM) of 24-h (24 h) heart rate (HR), muscle sympathetic nerve traffic (MSNA), and venous plasma norepinephrine (NE) in the groups of 62 healthy control subjects with resting 24-h heart rate < 70 beats minute, between 70 and 79 beats/minute and > 80 beats/minute. Bs/min indicates bursts/minute. Asterisks (*P < 0.05, **P < 0.01) refer to the statistical significance between groups

Individual and average clinic HR values assessed in the 3 groups are shown in Fig. 3, which also displays individual and average data obtained for 24-h HR Holter monitoring, MSNA and venous plasma NE. As expected, resting HR values were progressively and significantly greater from the group with an HR below 70 beats/minute to the ones displaying HR between 70 and 79 and above 80 beats/minute (Fig. 3, left upper panel). This was the case also for 24-h HR values (Fig. 3, left lower panel). At variance from what it has been reported above for HF patients, however, only subjects with clinic HR values greater than 80 beats/minute were characterized by MSNA values significantly increased when compared to those detected in HF with HR lower than 70 beats/minute (Fig. 3, upper middle panel), no significant difference being found for the group with HR between 70 and 79 beats/minute. A similar behavior was observed when HR values were assessed during the 24-h period (Fig. 3, lower middle panels). Again at variance from the findings we reported above in HF, no significant difference in plasma NE values was found between the 3 groups of healthy control individuals.

Similarly to what previously described for HF patients, in the whole healthy control population we found that MSNA and venous plasma NE showed a highly significant relationship with clinic HR, the correlation being much closer for MSNA (*r* = 0.71, *P* < *0*.0001) than for NE (*r* = 0.27, *P* < 0.03). Similar relationships were detected when 24-h HR values were taken into account. However, when correlations were sought separately in the 3 groups of healthy controls characterized by different HR values both MSNA and venous plasma NE failed to show any significant correlation with clinic or 24-h HR.

## Discussion

The present study provides a number of new information which can be summarized as follows. First, in HF patients clinic HR values above 70 beats/minute are accompanied by a sympathetic activation significantly and markedly greater for magnitude than the one detected in patients in which HF is associated with clinic HR values below 70 beats/minute. This means that this HR cutoff value applied in the SHIFT trial [4, 5], and the similar one used in other studies [6–10] is capable to detect HF patients with a lower or a higher neuroadrenergic cardiovascular drive. Second, the degree of sympathetic activation appears to closely mirror the HR increase found in the different HF groups. Third, this conclusion holds true not only when HR is measured in the clinical setting via the palpatory method but also when HR is evaluated via Holter monitoring during the 24 h. Fourth, the results are almost superimposable when sympathetic activity was directly quantified via the microneurographic technique as MSNA and when neuroadrenergic drive was assessed via a less direct approach, that is, the assay of venous plasma NE. Fifth, the study findings indicate that the assessment of adrenergic cardiovascular drive was, nevertheless, less sensitive when based on NE than on MSNA, this being particularly the case when subanalyses within each HF group were performed. Well defined are the pathophysiological and clinical implications of the present findings. Evidence is indeed available that in HF an increase in sympathetic drive to the heart and the peripheral circulation is accompanied by a greater risk of fatal and non-fatal cardiovascular events, the prognostic value of the sympathetic overactivity being shown to be independent on other confounders [11–18].

As reported above, in the present study we performed the above mentioned evaluation not only in HF patients but also in healthy controls. This allowed us determine whether the results we described in HF were specific for this clinical condition or rather they could be observed also in the general population as well. The results show that, at variance from HF patients, in healthy subjects the HR cutoff for identifying a significant increase in the different adrenergic markers we employed is positioned at a higher value, namely 80 beats/minute. This cutoff value thus appears to be superimposable to the one we found in a previous study in patients with essential hypertension [23]. Although the reasons responsible for the difference in the cutoff value between HF and essential hypertensive patients remain to be clarified, a likely hypothesis is that, because in HF the magnitude of the sympathetic overdrive is much greater than that found in healthy controls and in hypertensive patients, it can be more frequently detected even at lower HR values.

An additional result of our study concerns the finding that in healthy controls, but not in HF patients, venous plasma NE is unable to detect the differences in the sympathetic activity observed via microneurography in the subgroups of patients displaying progressively greater HR values. This finding is in line with the results of previous studies by our group and others showing the reduced sensitivity of plasma NE, compared to MSNA, in detecting changes in sympathetic activity [11, 22, 24]. Finally, the results of the present study provide evidence that at variance from what we found in each single subgroup of HF patients characterized by progressively greater HR values, in healthy controls neither NE assay nor a much sensitive marker of sympathetic drive such as MSNA was capable to display a significant relationship with HR.

### Limitations and clinical implications

Our study has some limitations but also clinical implications. One limitation refers to the retrospective nature of our study, which allowed us, however, to make use of one of the largest database available with the use of microneurographic nerve traffic recording together with venous plasma NE in assessing sympathetic drive in HF. A second limitation refers to the fact that due to ethical reasons we investigated HF patients under active pharmacological treatment with compounds capable to interfere with sympathetic cardiovascular regulation. It should be mentioned, however, that (1) the majority of HF patients evaluated in the present study were under treatment with a number of different compounds capable to trigger opposite sympathetic effects [18], thereby leaving, as a net result, sympathetic cardiovascular drive almost unaltered, and (2) beta-blocking drugs, i.e., the compounds directly acting on resting HR, were withdrawn 5–7 days prior to the study, allowing to rule out with a high probability any direct or indirect interference of these drugs with this hemodynamic variable as well as with the direct or indirect sympathetic indices employed in the present study.

The clinical implications are that the evidence that in HF patients showing resting HR greater than 70 beats/minute sympathetic activity is already markedly increased provides an important pathophysiologic background for defining patients at greater cardiovascular risk. The same HR cutoff value appears to be valid for employing in the therapeutic approach to HF drugs capable to exert sympathomoderating properties [25].

## Conclusions

In HF HR values greater than 70 beats/minute are associated with an increased sympathetic activation, both when assessed by direct recording of MSNA and when evaluated as plasma NE concentrations. The sympathetic overdrive parallels for magnitude the HR elevations, this being the case for both clinic and 24 h HR.

## Abbreviations

HF: Heart failure
HR: Heart rate
NE: Plasma norepinephrine
MSNA: Muscle sympathetic nerve traffic
