# Correction: Relationships between sympathetic markers and heart rate thresholds for cardiovascular risk in chronic heart failure

**DOI:** 10.1007/s00392-022-02128-6

**Published:** 2022-11-23

**Authors:** Guido Grassi, Gino Seravalle, Jennifer Vanoli, Rita Facchetti, Domenico Spaziani, Giuseppe Mancia

**Affiliations:** 1 Clinica Medica, Department of Medicine and Surgery, University of Milano-Bicocca, Via Pergolesi 33, 20052 Milan, Monza Italy; 2 IRCSS Istituto Auxologico Italiano, Milan, Italy; 3 Unità Operativa Complessa Di Cardiologia, Magenta Hospital, Milan, Magenta Italy; 4 University Milano-Bicocca, Milan, Italy

**Correction: Clinical Research in Cardiology** 10.1007/s00392-022-02028-9

The affiliation of the author Gino Seravalle was corrected to 'IRCSS Istituto Auxologico Italiano, Milan, Italy'. The original article has been corrected.

